# Genomic Signatures Predict Poor Outcome in Undifferentiated Pleomorphic Sarcomas and Leiomyosarcomas

**DOI:** 10.1371/journal.pone.0067643

**Published:** 2013-06-25

**Authors:** Sara Martoreli Silveira, Rolando Andre Rios Villacis, Fabio Albuquerque Marchi, Mateus de Camargo Barros Filho, Sandra Aparecida Drigo, Cristovam Scapulatempo Neto, Ademar Lopes, Isabela Werneck da Cunha, Silvia Regina Rogatto

**Affiliations:** 1 Neogene Laboratory, A. C. Camargo Cancer Center, São Paulo, São Paulo, Brazil; 2 Institute of Mathematics and Statistics, Inter-Institutional Program on Bioinformatics, USP, São Paulo, São Paulo, Brazil; 3 Department of Urology, Faculty of Medicine, UNESP, Botucatu, São Paulo, Brazil; 4 Department of Pathology, Barretos Cancer Hospital (Pio XII Foundation), Barretos, São Paulo, Brazil; 5 Department of Pelvic Surgery, A. C. Camargo Cancer Center, São Paulo, São Paulo, Brazil; 6 Department of Pathology, A. C. Camargo Cancer Center, São Paulo, São Paulo, Brazil; Utrecht University, Netherlands

## Abstract

Undifferentiated high-grade pleomorphic sarcomas (UPSs) display aggressive clinical behavior and frequently develop local recurrence and distant metastasis. Because these sarcomas often share similar morphological patterns with other tumors, particularly leiomyosarcomas (LMSs), classification by exclusion is frequently used. In this study, array-based comparative genomic hybridization (array CGH) was used to analyze 20 UPS and 17 LMS samples from untreated patients. The LMS samples presented a lower frequency of genomic alterations compared with the UPS samples. The most frequently altered UPS regions involved gains at 20q13.33 and 7q22.1 and losses at 3p26.3. Gains at 8q24.3 and 19q13.12 and losses at 9p21.3 were frequently detected in the LMS samples. Of these regions, gains at 1q21.3, 11q12.2-q12.3, 16p11.2, and 19q13.12 were significantly associated with reduced overall survival times in LMS patients. A multivariate analysis revealed that gains at 1q21.3 were an independent prognostic marker of shorter survival times in LMS patients (HR = 13.76; *P* = 0.019). Although the copy number profiles of the UPS and LMS samples could not be distinguished using unsupervised hierarchical clustering analysis, one of the three clusters presented cases associated with poor prognostic outcome (*P = *0.022). A relative copy number analysis for the *ARNT*, *SLC27A3,* and *PBXIP1* genes was performed using quantitative real-time PCR in 11 LMS and 16 UPS samples. Gains at 1q21-q22 were observed in both tumor types, particularly in the UPS samples. These findings provide strong evidence for the existence of a genomic signature to predict poor outcome in a subset of UPS and LMS patients.

## Introduction

Sarcomas are a heterogeneous group of mesenchymal tumors that represent approximately 1% of cancers diagnosed in adults and 15% of childhood tumors [1]. Soft-tissue sarcomas (STSs) are classified into two categories. The first group includes tumors with non-pleomorphic morphologies, which are usually associated with genomic translocations and certain specific mutations, and tumors with pleomorphic morphologies, which are associated with complex chromosomal alterations and genomic instability [2]. Leiomyosarcomas (LMSs) and undifferentiated high-grade pleomorphic sarcomas (UPSs) belong to the second STS group.

UPSs, which have been previously known referred to as malignant fibrous histiocytomas (MFHs), represent 5% of STSs diagnosed in adults [3]. Clinically, these aggressive tumors frequently show local recurrence and can metastasize to distant sites [4]. The absence of the lineage with specific differentiation observed in UPS reflects the difficulty of histopathological classification and the reproducibility of sarcoma diagnosis [5]. However, a number of important signaling pathways required for the maintenance of mesenchymal stem cells (MSCs) have been associated with UPS cell tumorigenicity [6], [7].

Most UPSs share similar morphologies with undifferentiated and pleomorphic tumor subtypes, particularly LMSs, liposarcomas, and rhabdomyosarcomas [4], [8]. LMSs represent more than 20% of STSs. Similar to UPSs, LMSs also display pleomorphic characteristics and often follow an aggressive course [9]. Several studies have evaluated gene-expression profiles from large STS cohorts, and they were unable to distinguish UPSs from LMSs based on hierarchical clustering analysis. However, in some cases, it was possible to identify minor UPS and LMS subgroups with similar gene-expression and/or genomic profiles [10], [11], [12], [13], [14].

DNA copy number profiles derived from UPS samples have revealed recurrent genomic alterations that are correlated with morphological subtypes and patient outcome. These genomic imbalances commonly include gains at the 17q locus, which have been associated with longer disease-free survival times and a lower risk of distant metastasis [15]. In addition, losses of 4q31 and 18q22 have been associated with an increased risk of metastasis and favorable prognosis in UPS and LMS, respectively [16]. Gains at 1p33-p32.3 and 1p21.3 in UPS have been recently associated with increased patient survival times [17]. Unfortunately, DNA copy number studies have evaluated small sample sizes. In addition, the majority of these reports have not described whether the evaluated UPS and LMS samples were obtained from treated or untreated patients. Importantly, accurate diagnoses are essential for these cancer types because distinct diagnostic entities may require different treatment strategies [10].

This study was designed to determine the potential of chromosomal imbalance profiles detected with array CGH methods to reveal biomarkers for diagnosis and/or prognosis. Additionally, the study aimed to identify novel putative molecular targets in untreated patients prior to surgery to improve therapies to treat UPS and LMS.

## Patients and Methods

### Patients

Thirty seven fresh frozen tissue samples (20 UPS and 17 LMS) were obtained from 36 patients who were followed prospectively at either A.C. Camargo Hospital (São Paulo, Brazil) or Barretos Cancer Hospital (Barretos, São Paulo, Brazil) between 2000 and 2010. The procedures were described to all of the patients, after which time they provided written informed consent. This study was approved by the Ethical Committee in Research of the Antonio Prudente Foundation at A.C. Camargo Hospital (Protocol 1105/08) and by the Ethical Committee in Research of the Pius XII Foundation at Barretos Cancer Hospital (Protocol 302/2010). The medical records of all of the patients were examined to obtain detailed demographic and clinicopathologic data (Table 1), and all of the cases were evaluated by an expert sarcoma pathologist (IWC). The diagnostic criteria were based on World Health Organization (WHO) recommendations and included both the morphology and expression of specific proteins detected using immunohistochemistry [18]. Histological grades were defined according to the recommendations of the Federation Nationale des Centres de Lutte Contre le Cancer (FNCLCC), which considers the mitotic index, tumor necrosis, and cell differentiation [19].

**Table 1:** Clinical and histopathological data from patients (20 UPS and 17 LMS).

Patient	Sample	Age	Sex	Sample origin	Location	TNM	Grade	Local Recurrence	Distance Metastasis	Treatment (QT or RT)	Follow-up (months)^c^
1	UPS1^d^ *	51	F	Primary tumor	Lower extremity	T1aN0M0	III	Presence	–	0	NED (108)
2	UPS2*	52	F	Recurrence	Retroperitoneum	T2bN0M0	III	Presence	–	2	DD (33)
3	UPS3^d^ *	49	M	Primary tumor	Retroperitoneum	T2bN0M0	III	Presence	–	2	DD (22)
4	UPS4*	90	M	Primary tumor	Lower extremity	T2aN0M0	III	–	–	2	DD (8)
5	UPS5^d^	50	M	Primary tumor	Retroperitoneum	T2bN0M0	III	Presence	–	2	DD (8)
6	UPS6	56	M	Primary tumor	Lower extremity	T2bN0M0	III	–	–	2	NED (44)
7	UPS7*	58	F	Recurrence	Head and Neck	T2aN0M0	III	Presence	–	2	LF (29)
8	UPS8^a.^ *	63	M	Primary tumor	Head and Neck	T1aN0M1	III	–	–	2	DD (15)
	UPS18^a^ *			Primary tumor	Lower extremity	T2bN0M1	III	–	Lung and adrenal (MD)	2	DD (7)
9	UPS9*	72	M	Primary tumor	Lower extremity	T2bN0M0	III	–	–	1	NED (30)
10	UPS13*	63	M	Recurrence	Lower extremity	T2bN0M0	III	Presence	Lung	1	DD (18)
11	UPS14*	32	F	Primary tumor	Trunk	T1aN0M0	III	–	–	0	NED (30)
12	UPS15*	80	M	Recurrence	Retroperitoneum	T2bN0M0	III	Presence	–	0	LF (11)
13	UPS16*	60	M	Primary tumor	Trunk	T2aN0M0	III	–	–	1	NED (1)
14	UPS17	56	M	Primary tumor	Lower extremity	T2bN0M0	III	–	–	1	NED (20)
15	UPS19	77	M	Primary tumor	Upper extremity	T2bN0M0	III	–	–	2	LF (33)
16	UPS20*	41	M	Primary tumor	Retroperitoneum	T2bN0M0	III	–	–	2	NED (6)
17	UPS21*	82	F	Primary tumor	Lower extremity	T2bN0M0	III	–	–	0	DD (3)
18	UPS22*	78	M	Primary tumor	Upper extremity	T2bN0M0	III	Presence	Lung	0	AD (11)
19	UPS23*	60	M	Recurrence	Lower extremity	T2aN0M0	III	Presence	Lung and bones	2	AD (5)
20	LMS3	60	F	Recurrence	Upper extremity	T1aN0M0	II	Presence	-	0	NED (64)
21	LMS4*	89	F	Recurrence	Trunk	T2bN0M0	II	Presence	Lung	0	DD (12)
22	LMS5	77	F	Recurrence	Trunk	T2bN0M0	III	–	Pelvis	0	DD (54)
23	LMS6	54	F	Primary tumor	Retroperitoneum	T2bN0M1	III	Presence	Liver (MD)	2	DD (15)
24	LMS7*	37	M	Primary tumor	Lower extremity	T1aN0M0	III	–	–	1	NED (45)
25	LMS8	61	F	Primary tumor	Retroperitoneum	T2bN0M0	III	–	Liver	0	DD (28)
26	LMS9	62	M	Primary tumor	Lower extremity	T2bN0M0	II	–	–	2	NED (99)
27	LMS15*	49	M	Primary tumor	Lower extremity	T2bN0M0	I	–	–	0	NED (65)
28	LMS16*	50	M	Recurrence	Retroperitoneum	T2bN0M0	III	Presence	–	0	NED (109)
29	LMS17	48	M	Primary tumor	Retroperitoneum	T2bN0M0	III	–	–	0	LF (4)
30	LMS18*	61	M	Primary tumor	Retroperitoneum	T2bN0M0	III	Presence	–	2	AD (36)
31	LMS19^b^ *	4	M	Primary tumor	Lower extremity	T2bN0M0	III	–	–	2	NED (25)
32	LMS20*	81	F	Primary tumor	Lower extremity	T2bN0M1	III	–	Lung, liver and abdominal wall (MD)	1	DD (10)
33	LMS21*	52	F	Primary tumor	Retroperitoneum	T2bN0M0	I	–	–	0	NED (23)
34	LMS22^b.^ *	74	F	Primary tumor	Retroperitoneum	T2bN0M0	II	–	–	3	LF (2)
35	LMS23*	45	F	Primary tumor	Retroperitoneum	T2bN0M0	III	Presence	–	0	NED (21)
36	LMS24*	58	F	Primary tumor	Upper extremity	T2aN0M0	III	–	–	2	NED (48)

Abbreviations - F: Female, M: Male, DD: Death by disease, NED: No evidence of disease, AD: Alive with disease, LF: Loss of follow-up; MD: metastasis at diagnosis.

Treatment – QT: Chemotherapy; RT: Radiotherapy; 0: Surgery; 1: Neoadjuvant therapy; 2: Adjuvant therapy; 3: Chemotherapy without surgery.

* Selected for qPCR validation.

a Samples from the same patient.

b Patients with Li-Fraumeni Syndrome.

c Time to last follow-up from diagnosis.

d Samples of different patients obtained from expansion of primary tumor surgical (remnant of primary tumor).

Twenty six out of 37 tumor samples (20 UPS and 17 LMS) were derived from primary tumors, eight from locally recurrent tumors and three from remnant tumors (derived of the surgical margins expansion from different patients). Two primary tumors (UPS8 and UPS18) were derived from the same patient (patient #8, Table 1). None of the patients had received chemotherapy or radiotherapy treatment prior to sample collection. One patient was diagnosed as a Li-Fraumeni Syndrome carrier (patient #34). The average patient age was 59.3 years (ranging from 4–90 years). The anatomical sites commonly affected were lower extremities (14 cases), retroperitoneum (13 cases), trunk (4 cases), upper extremities (4 cases) and head and neck (2 cases). According to the FNCLCC guidelines, the majority of the cases were classified as high histological grade (G2 or G3), and two of the LMS cases (LMS15 and LMS21) were classified as G1. Fourteen patients received only surgical treatment, six underwent neoadjuvant chemotherapy followed by surgery and 15 patients received adjuvant therapy after the surgery (including patient #8). One patient received only chemotherapy without surgery (patient #34). The mean follow-up time was 29.8 months (ranging from 1–109 months). In three patients distant metastases were detected at diagnosis (patients #8, #23 and #32).

A diverse panel of antibodies was used for the immunohistochemical characterization, including SMA (Cell Marque, clone 1A4), Desmin (Ventana, clone DER11), HHF35 (Cell Marque, clone HHF 35), Caldesmon (Dako, clone h-CD), CD34 (Ventana, clone QBEnd 10), CD31 (Ventana, clone JC70), CD99 (Ventana, clone 0.13), S100 (Ventana, clone PAB), NSE (Ventana, clone E27), AE1/AE3 (Ventana, pool), EMA (Ventana, clone E29), HMB45 (Ventana, clone HMB45), MART-1/MelanA (Ventana, clone A-103), CD45 (LCA) (Ventana, clone RP2/18), CD63 (Cell Marque, clone NK1/C3) and MDM-2 (Neomarkers, polyclonal). These markers were used to confirm or exclude other diagnoses, such as melanoma, lymphoma, or undifferentiated tumors. Cases were considered to be pleomorphic sarcomas when tumors showed pleomorphic morphology and were negative for all of the tested markers or when they presented focal expression of the muscle markers SMA, HHF35, Desmin, and/or Caldesmon. Tumors with spindle cell morphology and diffuse expression of muscle markers (SMA, HHF35, Desmin, or Caldesmon) were considered to be LMSs. Cases showing both spindle cell morphology and pleomorphic morphology in addition to the strong or diffuse expression of muscle markers (SMA, HHF35, Desmin, or Caldesmon) were also considered to be LMSs. The retroperitoneal UPS were carefully revised by two pathologists (IWC and CSN) with the aim to exclude occult cases of undifferentiated liposarcoma. Negative staining for MDM2 at immunohistochemistry and absence of any region with differentiated liposacoma features kept the diagnosis at UPS upon morphological grounds. In addition, FISH analysis was performed to investigate *MDM2* amplification in a subgroup of retroperitoneal sarcomas (3 UPS and 3 LMS) (Text S1). None of these cases had detectable *MDM2* amplification (Kreatech MDM2/CEN12, Amsterdam, ND) (Figure A and Table A in Text S1).

### Array-based Comparative Genomic Hybridization (array CGH)

Genomic DNA was extracted using a standard phenol/chloroform-based method. Genomic DNA samples from tumors and normal tissue (Promega, Madison, WI, USA) were differentially labeled using the Genomic DNA Enzymatic Labeling Kit (Agilent Technologies, Santa Clara, CA, USA). The hybridizations were performed on Agilent Human CGH 44 K Oligo Microarrays according to the manufacturer’s recommendations. The array images were acquired with a DNA microarray scanner using SureScan High-Resolution Technology and the Scan Control (version 8.1) software program (Agilent Technologies, Santa Clara, CA, USA). The data were analyzed using the Nexus Copy Number (version 6.0, Biodiscovery Inc., El Segundo, CA, USA) software program [20]. The Fast Adaptive States Segmentation Technique 2 (FASST2) algorithm and the Significance Testing for Aberrant Copy number (STAC) statistical method were used to identify non-random genomic copy number alterations [21]. Based on these algorithms, DNA copy number alterations were defined as instances that exceeded a significance threshold of 1×10^−5^ and that contained at least five consecutive altered probes per segment. These parameters were used to define the following: copy number gain (≥0.2), high copy number gain (≥0.6), copy number loss (≤0.2), and homozygous loss (≤−1.0). Genomic data discussed in this publication have been deposited in NCBI’s Gene Expression Omnibus and are accessible through GEO Series accession number GSE45573 (http://www.ncbi.nlm.nih.gov/geo/query/acc.cgi?acc=GSE45573).

### Quantitative Real-Time PCR (qPCR)

The genomic DNA sequences of candidate regions were obtained from the Ensembl Genome Browser website (GRCh37/hg19 Human Reference Assembly; February 2009). Primer sequences were designed using the Primer-Blast online software tool (http://www.ncbi.nlm.nih.gov/tools/primer-blast/). Eight primer pairs (*ARNT-*P1, *ARNT-*P2, *ARNT-*P3, *PBXIP1-*P1, *PBXIP1-*P2, *SLC27A3-*P1, *CCND1*-P1, and *CCND1-*P2) were designed to amplify the altered regions detected using array CGH, including the 60-nucleotide probe present on the Agilent platform (Table S1). Standard curves generated to ensure optimal amplification efficiency (90–100%) were created using five template concentrations from four-fold serial dilutions (ranging from 80–0.31 ng). The reactions were carried out by automated pipetting using the QIAgility system (Qiagen, Courtaboeuf, France) in a total volume of 12.5 µl. Each reaction contained Power SYBR Green PCR Master Mix (Applied Biosystems, Foster City, CA, USA), 20 ng of DNA, and 200 nM of each primer. The reactions were performed in duplicate, and the following PCR cycling conditions were used: an initial hold at 95°C for 10 min and 40 cycles of 95°C for 15 s and 58–59°C for 1 min. A dissociation curve was performed after the amplification cycle using the 7500 Real-Time PCR System (Applied Biosystems, Foster City, CA). The specificity of the amplified products was verified by analyzing the dissociation curves and the variation between replicates. Any instances in which the cycle quantification (Ct) values were greater than 0.5 were reassessed.

By qPCR analyses, DNA samples from 10 healthy individuals (reference controls) were compared with 11 LMS and 16 UPS samples (previously evaluated by array CGH). The relative copy numbers were calculated according to the delta-delta Ct model [22] using *GAPDH* as the reference gene. The relative copy number was calculated based on the target gene/*GAPDH* ratio, and this value was defined as a loss when the ratio was <0.55 and as a gain when the ratio was >1.35 (based on reference intervals).

### Statistical Analyses

Comparisons between groups with clinicopathological and molecular alterations were performed using the Fisher exact test and Student’s t-test. Overall survival (OS) probabilities were calculated using the Kaplan-Meier method and the Log Rank test for significance. The end-point for the OS analysis was restricted to deaths due to cancer. A multivariate analysis was performed using Cox proportional hazards with a model that included significant chromosomal alterations in LMS, tumor size, topography, tumor depth, local recurrence, and treatment (i.e., chemotherapy and/or radiotherapy). Statistical analyses were carried out using the software programs Nexus Copy Number (version 6.0; Biodiscovery Inc., El Segundo, CA, USA), Graphpad Prism 5 (Graphpad Software Inc., La Jolla, CA, USA), and SPSS version 17.0 (SPSS, Chicago, Illinois, USA) for Windows.

## Results

### Genome-wide Profiling of UPS and LMS

Several genomic changes were detected in the UPS and LMS samples, with UPS showing more complex genomic alterations. Changes in DNA copy number were identified in more than 20% of the UPS and LMS cases (*P*<0.05) as shown in Table 2.

**Table 2:** Genomic imbalances more frequently detected in UPS and LMS.

Cytoband location	Start (bp)	Stop (bp)	Size (Mb)	Event	Genes	miRNAs	Frequency (%)	P-Value
**UPS**								
1q21.1-q21.2	147,458,669	149,236,666	1.8	Gain	64	0	40.0	0.004
1q21.3-q23.1	151,345,357	155,272,136	3.9	Gain	186	5	60.0	0.004
2q11.1-q11.2	95,562,577	98,202,102	2.6	Gain	53	0	45.0	0.013
3p26.3	0	726,469	0.7	Loss	4	0	60.0	0.015
3p12.1-p11.2	85,966,634	87,627,650	1.7	Gain	7	0	30.0	0.009
7q22.1	99,861,211	100,667,677	0.8	Gain	61	0	60.0	0.042
8p11.21	41,627,121	43,175,310	1.5	Gain	25	1	40.0	0.032
9q34.11	129,506,829	130,229,037	0.7	Gain	49	2	45.0	0.008
11p15.5	0	2,197,662	2.2	Gain	118	3	45.0	0.020
11q13.1	65,171,847	66,296,450	1.1	Gain	60	0	40.0	0.021
11q13.1-q13.2	66,882,158	67,549,110	0.7	Gain	36	0	30.0	0.021
16p13.3	1,062,920	1,341,725	0.3	Gain	14	0	40.0	0.036
16q24.3	87,762,155	88,283,196	0.5	Gain	22	0	20.0	0.020
18p11.32	0	846,102	0.8	Gain	12	0	35.0	0.034
20p11.21	22,798,105	23,302,271	0.5	Gain	10	0	45.0	0.007
20q13.33	60,236,430	61,684,607	1.4	Gain	64	3	75.0	0.004
**LMS**								
1q21.3	151,256,551	151,856,750	0.6	Gain	27	0	23.5	0.018
1q21.3-q22	152,467,522	154,387,590	1.9	Gain	88	2	23.5	0.018
6p21.32	31,923,769	32,132,073	0.2	Gain	24	1	23.5	0.008
7q22.1	99,647,068	100,857,537	1.2	Gain	78	0	29.4	0.006
8q24.3	143,523,381	146,274,826	2.8	Gain	127	4	47.0	0.045
9p21.3	21,199,776	22,226,425	1.0	Loss	23	1	41.2	0.023
11p15.5	0	1,567,792	0.9	Gain	87	1	23.5	0.011
11q12.2-q12.3	61,352,779	62,229,449	0.9	Gain	37	0	29.4	0.047
11q13.1-q13.2	64,322,265	67,979,861	3.7	Gain	181	3	29.4	0.047
11q13.2-q13.3	68,744,800	70,077,870	1.3	Gain	19	1	29.4	0.047
14q11.2	21,580,291	22,230,297	0.7	Gain	44	0	23.5	0.007
16p11.2	34,059,589	34,361,998	0.3	Loss	1	0	23.5	0.031
17q25.1	68,836,129	70,494,896	1.7	Gain	40	0	41.2	0.050
19q13.12	40,964,925	41,016,382	0.1	Gain	5	0	52.9	0.008
19q13.43	63,347,573	63,811,651	0.5	Loss	36	0	23.5	0.043

Legend: UPS - Undifferentiated Pleomorphic Sarcomas; LMS - Leiomyosarcomas.

The most frequently observed significant alterations in the UPS cases involved gains at 20q13.33 (75% of cases); 1q21.3-q23.1 (60%); 7q22.1 (60%); 9q34.11 and 20p11.21 (45%); and 1q21.1-q21.2, 8p11.21, 11q13.1 and 16p13.3 (40%) (Table 2). Genomic amplification (log_2_ ratio >0.6) was observed at 1q21.1-q21.2 (UPS7), 1q21.3-q23.1 (UPS7 and UPS8), 3p12.1-p11.2 (UPS2, UPS9, UPS15, UPS19, and UPS22), 7q22.1 (UPS4), 8p11.21 (UPS22), 11q13.1 (UPS3 and UPS8), 16p13.3 (UPS2), 18p11.32 (UPS13), and 20p11.21 (UPS19 and UPS22). Losses at 3p26.3 were observed in 60% of the cases (Table 2). No homozygous deletions (log_2_ ratio<−1) were consistently observed in the UPS cases. None of these UPS alterations were significantly associated with clinical variables.

The LMS samples showed fewer genomic alterations compared with the UPS samples. Fifteen significant genomic imbalances were more frequently observed in the LMS samples (Table 2), the most notable being gains at 19q13.12 (53%) and 8q24.3 (47%). Losses at 9p21.3 (*P = *0.023) and gains at 17q25.1 (*P = *0.050) were observed in 41.2% of the samples, including two cases (LMS19 and LMS23) with homozygous deletions (log2 ratio<−1) at 9p21.3.

**Figure 1:**
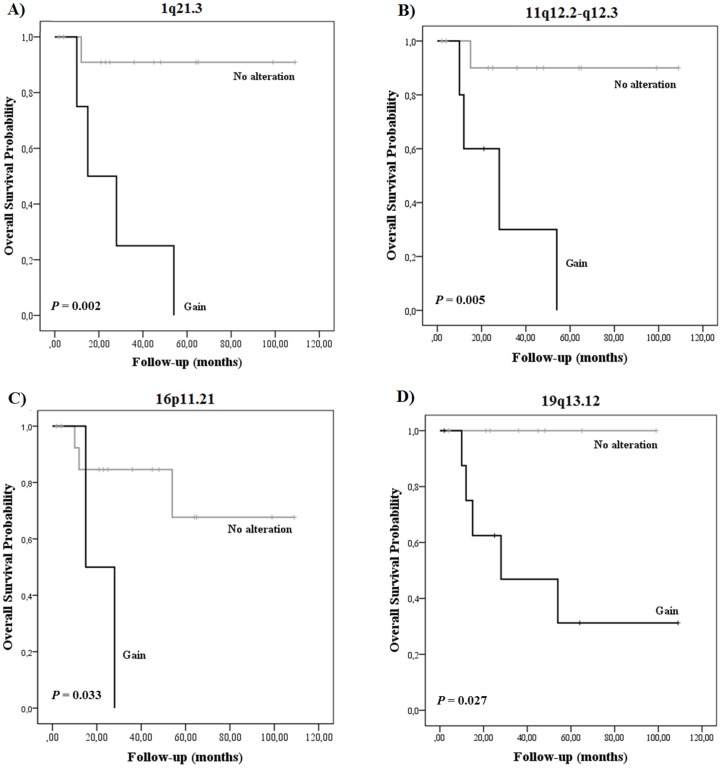
Overall survival curves from LMS patients with specific genomic alterations. Gains at (A) 1q21.3, (B) 11q12.2-q12.3, (C) 16p11.2, and (D) 19q13.12 were associated with shorter survival times. P-values were determined using the Log-rank test.

Gains at 1q21.3, 11q12.2-q12.3, and 19q13.12 were significantly associated with death caused by LMS (Table 3). Furthermore, reduced overall survival time was significantly associated with gains at 1q21.3 (*P = *0.002), 11q12.2-q12.3 (*P = *0.005), 16p11.2 (*P = *0.033), and 19q13.12 (*P = *0.027) (Figures 1A–D; Table 3). A multivariate analysis indicated that gains at 1q21.3 are an independent prognostic marker for shorter overall survival time (*P* = 0.019; HR = 13.76; CI_95%_ = 1.534 to 123.427).

**Table 3:** Genomic alterations associated with clinical outcome in LMS patients.

Chromosome region	Alteration	Prognosis*	Survival**
1q21.3	Gain	Increased risk of death (P = 0.002)	Decreased overall survival (P = 0.002)
11q12.2-q12.3	Gain	Increased risk of death (P = 0.0099)	Decreased overall survival (P = 0.005)
16p11.2	Gain	Increased risk of death (P = 0.073)	Decreased overall survival (P = 0.033)
19q13.12	Gain	Increased risk of death (P = 0.003)	Decreased overall survival (P = 0.027)

* P values obtained by Fisher’s exact test.

** P values obtained by log-rank test.

Although the majority of the UPS and LMS tumors were found in the retroperitoneum and lower extremities, two LMS samples preferentially localized to the trunk were characterized by significant recurrent alterations, including gains at 6p21.32 (*P = *0.044), 14q11.2 (*P = *0.044), 17q25.1 (*P = *0.029), and 19q13.43 (*P = *0.007).

**Figure 2:**
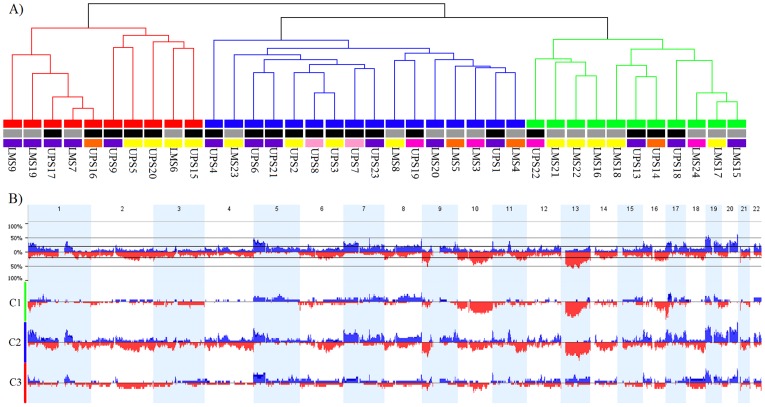
Unsupervised hierarchical clustering of 20 undifferentiated pleomorphic sarcomas (UPSs) and 17 leiomyosarcomas (LMSs). (A) In the dendrogram, cluster 1 is shown in green, cluster 2 is shown in blue, and cluster 3 is shown in red. Clusters related to the sites of anatomical origin were not observed for these tumors; origin sites include the following regions: upper extremity (pink), lower extremity (purple), trunk (orange), retroperitoneum (yellow), and head and neck (rose). (B) Genomic alterations were detected in clusters 1 (C1; 11 cases), 2 (C2; 16 cases), and 3 (C3; 10 cases). The top bars (blue) indicate genetic gains, whereas the lower bars (red) indicate genetic losses. The images shown were adapted from the output of the Nexus 6.0 software program.

An unsupervised hierarchical clustering analysis could not distinguish between the UPS and LMS samples, nor could it segregate the samples according to anatomical origin; however, three different clusters (1–3) were observed (Figures 2A and B). Similar numbers of genomic alterations were observed in clusters 1 (4 UPS and 7 LMS) and 3 (6 UPS and 4 LMS), whereas cluster 2 (10 UPS and 6 LMS) exhibited a more complex genomic profile (Figure 2B). Furthermore, the chromosomal changes in these three clusters were correlated with the clinicopathological features of each patient (Table 1). The presence of genomic alterations in cluster 2 was significantly correlated with female patients (*P = *0.020) and with death from UPS or LMS (*P = *0.022). Furthermore, cluster 2 included 70% of the cases (5/8 UPS and 4/5 LMS) in which patients died from the disease and 62% (3/5 UPS and 2/3 LMS) of the cases in which patients developed metastases during the follow-up period. Cluster 3 was primarily composed of male patients (*P* = 0.023). Interestingly, gains at 1q21.3 were more frequently detected in cluster 2 (62.5%) compared with clusters 3 (30%) and 1 (0%) (*P = *0.0035).

### Quantitative Analysis of Copy Number Alterations in UPS and LMS

A subset of the cases (11 LMS and 16 UPS; Table 1) was evaluated by qPCR to confirm the gains at 1q21.1-q21.2 (*ARNT*), 1q21.3 (*PBXIP1* and *SCL27A3*), and 11q13.2-q13.3 (*CCND1*). Eight primer pairs were designed to cover the candidate regions. Three primer pairs covered the same probe sequence included in the Agilent 4×44 K platform (*ARNT*-P1, *PBXIP1*-P2 and *CCND1*-P2), whereas two primer pairs flanked the specific probes to determine the extent of the alteration (*SLC27A3*-P1 and *ARNT*-P2). Additionally, other three primers pairs were also designed in regions of the exon-intron junctions (*ARNT*-P3, *PBXIP1*-P1, and *CCND1*-P1). The ten reference samples isolated from healthy individuals displayed normal copy numbers for each of the sequences evaluated by qPCR.

**Figure 3:**
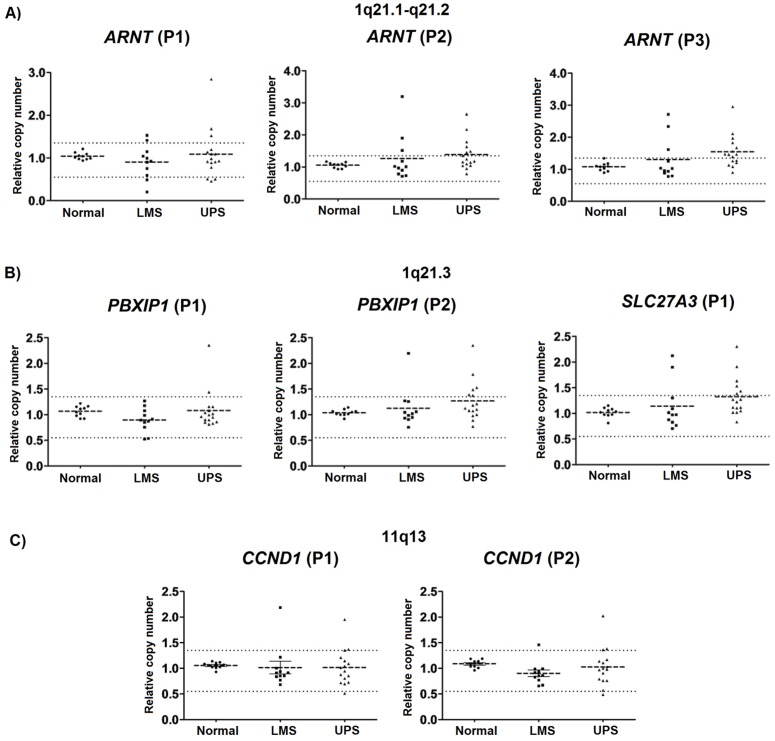
Quantification of DNA copy number alterations using qPCR for the *ARNT*, *PBXIP1, SLC27A3,* and *CCND1* genes. Eight primer pairs were designed, including (A) three for *ARNT* (ARNT-P1, ARNT-P2, and ARNT-P3); (B) two for *PBXIP1* (PBXIP1-P1 and PBXIP1-P2) and one for *SLC27A3* (SLC27A3-P1); and (C) two for *CCND1* (CCND1-P1 and CCND1-P2).

For *ARNT* region, relative DNA copy number gains were observed in both UPS and LMS, but these genomic alterations were most frequently observed in UPS (10/16 samples) compared with LMS (4/11 samples). Three UPS (UPS7, UPS8, and UPS9) and two LMS (LMS4 and LMS7) samples showed gains over a large region (17 kb) surrounding the *ARNT* gene (for all primer pairs) (Figure 3A, Table S2). In addition, seven UPS and two LMS samples showed gains at two primer sets (Table S2). Similarly, gains at *PBXIP1* were more frequently detected in UPS (6/16) than in LMS (1/11) samples. Two UPS (UPS7 and UPS9) showed gains in both primer sets flanking *PBXIP1*, which span a region covering approximately 2 kb (Table S2). For *SLC27A3* gene, 6 UPS and two LMS cases exhibited increased copy numbers (Figure 3B, Table S2).

The *ARNT, PBXIP1* and *SCL27A3* genes, which are mapped to 1q21, cover a chromosomal region of approximately 4.12 Mb. Copy number gains involving these three genes were detected in two UPS cases (UPS7 and UPS9, Table S2), one of which displayed amplification (UPS9), whereas the other (UPS7) exhibited complex morphology.

Interestingly, the proportion of cases with concordant results of copy number gains in UPS samples detected by both array CGH and qPCR was 83% (5/6) for 1q21.1-q21.2 (*ARNT*) region and 63% (7/11) for 1q21.3 9 (*PBXIP1* and S*CL27A3*). For *CCND1* gene (11q13), copy number gains in both primer sets were detected in three UPS samples (UPS2, UPS20, and UPS22), suggesting that at least 5 kb of genomic sequence was altered in these samples. Two LMS samples showed increased copy number for one or more of the sequences amplified by each primer pair (P1 and P2) (Figure 3C, Table S2).

## Discussion

In general, UPSs and LMSs display similar profiles of recurrent chromosomal imbalances even when compared with other sarcoma subtypes [9], [12], [14], [16], [17], [23], [24]. Some studies have suggested that the similarities shared by UPSs and LMSs may indicate a common origin [16], [25], [26], [27]. However, the majority of these studies did not report whether the tested samples were collected from patients who had received either chemotherapy or radiotherapy prior to surgery. In this study, none of the patients had received treatment prior to sample collection, thereby excluding the possibility of treatment-induced genetic changes.

UPS and LMS frequently show complex karyotypes, pleomorphic histology and undifferenciated molecular profiles, thus hindering the correct diagnosis and the development of therapeutic options for these patients. Consequently, studies addressing the identification of molecular events driving oncogenesis in these tumors can ultimately be translated into meaningful biomarkers [18]. To address this challenge, we performed a comparative analysis of genomic copy number profiles for UPS and LMS samples carefully diagnosed by combining histology evaluation, immunohistochemical characterization using a panel of antibodies as well as FISH analysis for *MDM2*. Although an unsupervised hierarchical clustering analysis could not distinguish between the tumors subtypes, three patient clusters were identified based on patterns of copy number alterations. Of these three clusters, cluster 2 (16 UPS and 10 LMS) was strongly associated with poor prognostic outcome. More specifically, 105 genomic alterations were exclusively observed in cluster-2 cases (>50% of cases), including gains at 1q21.2, 1q21.3, 9q34.11, 11p15.5, 11q13.1, 16p13.3, and 20q13.33. Notably, gains at 1q21.3 in samples from cluster 2 were significantly associated with poor prognostic outcome.

The genetic similarities observed between UPS and LMS may indicate a common origin for these tumor subtypes. This hypothesis has also been raised by other comparative studies that assessed genomic patterns (using chromosomal CGH and array CGH) and gene-expression profiles (using cDNA microarrays and RT-qPCR) in UPS and LMS [16], [17], [23], [24], [25]. For example, Kresse et al. [17] compared genomic gains and losses in 33 UPS and 44 LMS samples using array CGH analysis (BAC and PAC arrays of 4.6K). The authors reported seven chromosomal regions (1p36.32-p35.2, 1p21.3-p21.1, 1q32.1-q42.13, 2q14.1-q22.2, 4q33-q34.3, 6p25.1-p21.32, and 7p22.3-p13) that differed significantly between the UPS and LMS tumor subtypes; three of these regions (1p35.1-p36.32, 1q32.1-q42.13, and 7p22.3-p13) were also identified in this study.

In another study using integrative analyses, similar patterns of genomic alterations were observed in 18 UPS and 31 LMS samples taken from tumors of the extremities [16]. The gene-expression analysis revealed nine differentially expressed genes (*TAGLN3, D4S234E, KIAA1729, PDLIM5, TEAD3, TPM2, ALDH1B1, TRDMT1,* and *DHODH*) but failed to differentiate between tumor subtypes. In the current study, these genes were not observed among the recurrent genomic regions that were differentially altered. Larramendy et al. [27] analyzed 102 untreated primary UPS samples and 82 LMS samples using chromosomal CGH, and the authors reported similar profiles of genetic alterations between these two tumor subtypes. Although a clustering analysis could not differentiate between the UPS and LMS samples, the authors reported one cluster (2 LMS and 10 UPS) that was characterized by high-level amplifications of the 1p33-p34.3, 17q22-q23, 17q25-qter, 19p, 22p, and 22q loci. Similarly, we observed genetic amplification at 17q25, 19p, and 22q; however, these alterations were not restricted to one specific cluster.

Large genomic regions (up to 4 Mb) displaying changes in DNA copy number were detected in both UPS and LMS samples. To better characterize these alterations, four genes (*ARNT, PBXIP1, SLC27A3,* and *CCND1*) were selected and evaluated using qPCR in a subset of cases. In general, the alterations detected by array CGH in the UPS and LMS groups were confirmed by qPCR. Although *ARNT* copy number gains were observed in both tumor subtypes, these alterations were predominantly observed in the UPS samples (63%). Several studies have demonstrated that amplification of the 1q21-q22 locus occurs in UPS and a variety of other STSs, including liposarcomas and osteosarcomas [15], [17], [28], [29], [30], [31], [32], [33]; however, no specific candidate genes from this region had been studied in the context of UPS. Aryl hydrocarbon receptor nuclear translocator (*ARNT*), which is also known as hypoxia-induced factor-1 beta (HIF-1beta), is constitutively expressed in all normal human tissues with increased expression in the ovary, lung, spleen, testis, and pancreas [34]. *ARNT* overexpression has been reported in breast cancer, hepatocellular carcinoma, and colon carcinoma cell lines [35], [36]. *PBXIP1* and *SLC27A3* copy number gains were also observed in UPS samples (38% of the cases for each). The *SLC27A3* gene encodes Acetyl-CoA synthetase, which is important for fatty-acid metabolism, particularly in neoplastic cells [37]. Although the contribution of lipid-metabolic pathways to tumor development is poorly understood, it is known that a high rate of lipid synthesis is necessary for the biogenesis of plasma membranes, which is required for tumor growth [38]. Lipids also play important roles as second messengers, which can be misregulated in tumor cells. Indeed, increases in the levels of specific messenger lipids are often associated with malignant phenotypes [39]. Functional studies have shown *SLC27A3* to be an effective therapeutic target in gliomas because it maintains the oncogenic properties of glioma cell lines through the regulation of the AKT protein [37].

Sixteen recurrent genomic changes were identified in UPSs. Although none of these changes were significantly associated with clinical variables, high-level amplification of the 3p12.1-p11.2 locus was observed in five cases. This amplified region spans the seven following genes: *BC040985, BC050344, CADM2, CHMP2B, MIR4795, POU1F1,* and *VGLL3.* Consistent with our findings, Hallor et al. [40] demonstrated that amplification of the 3p11-12 region was associated with *CHMP2B* and *VGLL3* overexpression in UPS cases with prominent inflammation. In another study, Carneiro et al. [16] identified recurrent amplifications at 3p11-p12 in a subset of UPS and LMS tumors using genomic and transcriptomic analyses. These findings suggest that the 3p11-p12 region, which includes the *CHMP2B* and *VGLL3* genes, may play an important role in UPS and LMS tumors.

Among the 15 minimally recurrent altered regions identified in the LMS samples, four regions showing gains were significantly associated with reduced overall survival time (1q21.3, 11q12.2-q12.3, 16p11.2, and 19q13.12). To our knowledge, this signature of poor prognosis has not been previously reported for LMS. In our study, copy number gains at 11q12.2-q12.3 and 19q13.12 were associated with death from LMS. The 11q12.2-q12.3 locus contains 37 genes, including genes related to the processes of chromosome segregation (*INCENP*), chromatin remodeling and histone deacetylation (*MTA2*), transcriptional regulation (*EEF1G*), and RNA processing (*TUT1*). To date, these genes have not been linked to LMS, and they represent potential targets for further validation. For example, metastatic tumor antigen 2 (MTA2) is a member of the MTA family and is closely associated with tumor progression and metastasis. MTA2 overexpression has been correlated with advanced TNM stages, tumor size, and lymph-node metastasis in non-small-cell lung cancer [41]. Gains at 19q13.12 have been described in sporadic cases using CGH approaches without clinical association [25], [42], [43]. We report an association between cases with poor prognosis and genomic gains at 19q13.12, indicating that this region may be a useful marker for LMS outcome. Furthermore, this amplified region has been linked to several cancer types, including pancreatic carcinoma [44], ovarian carcinoma [45], and breast cancer [46].

Genomic gains at 1q21.3 were associated with reduced overall survival time in LMS. A multivariate analysis revealed that gains at the 1q21.3 locus were an independent prognostic marker of shorter survival times for LMS patients. These data suggest that gains at 1q21.3 confer an increased risk of death from the disease compared with other genomic changes and known prognostic factors in STSs. Although increased copy numbers and high-level amplification of 1q21.3 have been frequently observed in chromosomal and array CGH studies of LMS samples [16], [28], [42], [47], [48], none have reported an association with poor prognosis. This region covers 27 genes, including *MUC1, SPRR1B, SPRR2A, SPRR3, RAB25,* and 13 members of the S100 family. Of these genes, low amplification levels of *MUC1, SPRR1B, SPRR2A, SPRR3,* and *S100A6* (mapped to 1q21-q22) have been observed in LMS samples using FISH analysis [28]. In addition to *RAB25* amplification, rearrangements and increased expression of the *S100A4* gene have been correlated with advanced disease stages and poor survival in other malignancies, such as ovarian osteosarcoma metastasis and ovarian carcinoma [49]. Further analysis of gene-expression profiles and functional data should be conducted to determine whether these genes play a similar role in LMS.

In conclusion, we describe a large number of genomic changes observed in UPS and LMS patients who were not previously treated with chemotherapy or radiotherapy. Importantly, a subset of patients with poor prognosis displayed recurrent gains at the 1q21.2, 1q21.3, 9q33.3-q34.11, 11p15.5, 11q13.1, 16p13.3, and 20q13.33 loci. These loci may be useful as diagnostic markers to distinguish between patient outcomes. Three novel candidate genes associated with the amplified 1q21 region, including the *ARNT* gene, were identified in UPS patients. Gains at 1q21.3 were shown to be an independent prognostic marker for shorter survival times in LMS patients, suggesting that genes mapped to this region may be involved in the aggressiveness of LMS tumors. Therefore, this study describes several novel molecular markers that may be used to identify leiomyosarcoma patients with poor outcomes.

## Supporting Information

Table S1 Primer sets used by quantitative real time PCR for confirmation of DNA genomic imbalances in UPS and LMS cases.
(DOC)

Table S2 DNA copy number alterations detected by qPCR at *ARNT, PBXIP1, SLC27A3* and *CCND1* genes in 16 UPS and 11 LMS samples. Legends: DNA copy number alterations are shown in filled boxes, including gains (light gray) and high copy gain (dark gray). Empty boxes represent absence of alteration.
(DOC)

Text S1 Fluorescence in situ hybridization for *MDM2/CEN12.*
(DOCX)
