# Supplementary material for: Genomic Signatures Predict Poor Outcome in Undifferentiated Pleomorphic Sarcomas and Leiomyosarcomas
Source: PLoS One. 2013 Jun 25;8(6):e67643. doi: 10.1371/journal.pone.0067643 (PMC3692486; doi:10.1371/journal.pone.0067643)
Supplement: Table S1 — Primer sets used by quantitative real time PCR for confirmation of DNA genomic imbalances in UPS and LMS cases. (DOC) [file pone.0067643.s001.doc]

**Table S1.** Primer sets used by quantitative real time PCR for confirmation of DNA genomic imbalances in UPS and LMS cases.

| **Probe ID*** | **Primers Set** | **Primers Sequences** |
| --- | --- | --- |
| ***ARNT*** |  |  |
| A_14_P136306 | P 1 | 5’-GATAAGGAGCGGTTTGCCAGGTA-3’ |
|  |  | 5’-AGTCAATATGCTAGGACTGTCTGGT3’ |
| A_14_P138897 | P 2 | 5’-TCTCCCGACACAACATTGAGGGT-3’ |
|  |  | 5’-TGGTAGCCAACAGTAGCCACACA-3’ |
| A_14_P135828 | P 3 | 5’-ACCTGGATGGGGCTTCTCTTTCT-3’ |
|  |  | 5’-TAGGCTGTCATCTTGTTCCGTCG-3’ |
| ***PBXIP1*** |  |  |
| A_14_P200039 | P 1 | 5’-TCAGGGACCTCAGCAACTATGGC-3’ |
|  |  | 5’-GACCCACCAAGAAAGAAGCCCAG-3’ |
| A_14_P135389 | P 2 | 5’AGGGAAGGGTGGAGATTAGACAGCA-3’ |
|  |  | 5’-CACCTCTTCTCCCAAGCCAGCA-3’ |
| ***SLC27A3*** |  |  |
| A_14_P120830 | P 1 | 5’-GGTAAGCCAGCAGTCCCCATT-3’ |
|  |  | 5’-ACATCCTTTAGCAACTTCCCCTGG-3’ |
| ***CCND1*** |  |  |
| A_14_P124581 | P 1 | 5’-CGCCCTCGGTGTCCTACTTCA-3’ |
|  |  | 5’-TAAGAGAGCCGCCCGAAGCC-3’ |
| A_14_P102675 | P 2 | 5’-TGGGGTGTACTTGGTCTGTGCT-3’ |
|  |  | 5’-GCTTGGCTCACTCGGGAAGG-3’ |
