# Supplementary material for: Genomic Signatures Predict Poor Outcome in Undifferentiated Pleomorphic Sarcomas and Leiomyosarcomas
Source: PLoS One. 2013 Jun 25;8(6):e67643. doi: 10.1371/journal.pone.0067643 (PMC3692486; doi:10.1371/journal.pone.0067643)
Supplement: Table S2 — DNA copy number alterations detected by qPCR at ARNT, PBXIP1, SLC27A3 and CCND1 genes in 16 UPS and 11 LMS samples. Legends: DNA copy number alterations are shown in filled boxes, including gains (light gray) and high copy gain (dark gray). Empty boxes represent absence of alteration. (DOC) [file pone.0067643.s002.doc]

**Table S2.** DNA copy number alterations detected by qPCR at *ARNT, PBXIP1, SLC27A3* and *CCND1* genes in 16 UPS and 11 LMS samples.

| **Sample** | **1q21.1-q21.2** | | | **1q21.3** | | **1q21.3** | **11q13.2-q13.3** | |
| --- | --- | --- | --- | --- | --- | --- | --- | --- |
|  | *ARNT-P1* | *ARNT-P2* | *ARNT-P3* | *PBXIP1-P1* | *PBXIP-P2* | *SLC27A3-P1* | *CCND1-P1* | *CCND1-P2* |
| UPS2 |  |  |  |  |  |  |  |  |
| UPS3 |  |  |  |  |  |  |  |  |
| UPS4 |  |  |  |  |  |  |  |  |
| UPS7 |  |  |  |  |  |  |  |  |
| UPS8 |  |  |  |  |  |  |  |  |
| UPS9 |  |  |  |  |  |  |  |  |
| UPS13 |  |  |  |  |  |  |  |  |
| UPS16 |  |  |  |  |  |  |  |  |
| UPS20 |  |  |  |  |  |  |  |  |
| UPS21 |  |  |  |  |  |  |  |  |
| UPS22 |  |  |  |  |  |  |  |  |
| UPS23 |  |  |  |  |  |  |  |  |
| LMS4 |  |  |  |  |  |  |  |  |
| LMS7 |  |  |  |  |  |  |  |  |
| LMS18 |  |  |  |  |  |  |  |  |

Legends: DNA copy number alterations are shown in filled boxes, including gains (light gray) and high copy gain (dark gray). Empty boxes represent absence of alteration.
