# Supplementary material for: Genomic Signatures Predict Poor Outcome in Undifferentiated Pleomorphic Sarcomas and Leiomyosarcomas
Source: PLoS One. 2013 Jun 25;8(6):e67643. doi: 10.1371/journal.pone.0067643 (PMC3692486; doi:10.1371/journal.pone.0067643)
Supplement: Text S1 — Fluorescence in situ hybridization for MDM2/CEN12. (DOCX) [file pone.0067643.s003.docx]

**Text S1.** Fluorescence in situ hybridization for *MDM2/CEN12.*

Coated glass slides containing formalin fixed paraffin embedded tissues were constructed with 3-μm cross-sections for FISH analysis. Hybridization, suppression hybridization, detection and fluorescence microscopy were performed as previously described in 5 UPS (3 from retroperitoneum) and 6 LMS (3 from retroperitoneum) (Rogatto SR, Rainho CA, Zhang ZM et al. *Cancer Genet. Cytogenet.* 1999;110;23-27). Commercially fluorescently labeled DNA probes for MDM2 and centromere region (Kreatech, Amsterdam, ND) were hybridized according the manufacturer’s recommendations. Control hybridization to interphase nuclei was performed. No overlapping interphase nuclei with intact morphology based on 4,6-diamino-2-phenylindole (DAPI) counterstaining were scored to determine the number of hybridization signals for each target probe. A case was considered to present copy number gains when the percentage of the hybridization showed three signals per cell relative to the two centromere signals and showed a number higher than the mean plus two standard deviations (2 SD) obtained for the same chromosome in the normal control. Amplification was defined as more than five signals relative to the two centromere signals and present in more than 25% of the cells.

Three out of five 5 UPS evaluated by FISH were derived from retroperitoneum and 2 UPS were from other sites (detailed in Table A). FISH analysis showed *MDM2* gain in one case (UPS15) (Figure A). Although the UPS15 (from retroperitoneum) presented *MDM2* gains, it was not detected any area with convincing well-differentiated liposarcoma feature. As additional information, we evaluated *MDM2* copy number alterations in three LMS from retroperitoneum (LMS6, LMS16 and LMS22) and three from other sites (Table A). The three retroperitoneal LMS presented gains of chromosome 12 (4 copies of the *MDM2* gene).


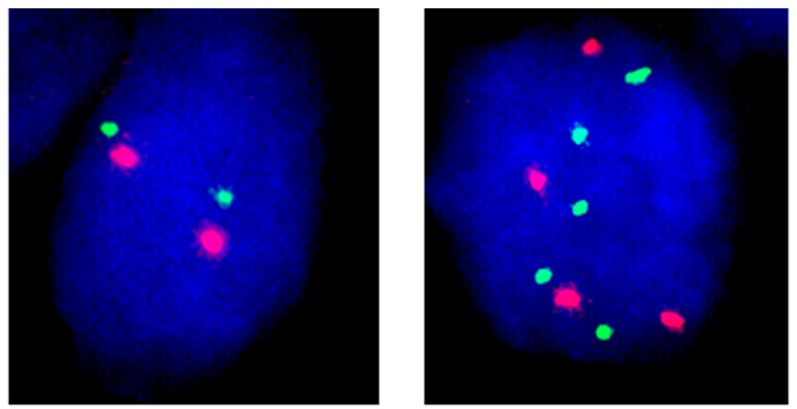


**Figure A.** Interphase nuclei hybridized with *MDM2/CEN12* (Kreatch, ND) showing normal pattern (two signals in green/CEN12 and two signals in red/*MDM2*) and gains of both probes in retroperitoneal UPS case.

**Table A.** *MDM2* copy number alterations by FISH analysis in UPS and LMS samples.

| **Sample ID** | **Location** | **FISH** |
| --- | --- | --- |
| **UPS2** | Retroperitoneum | Normal |
| **UPS3** | Retroperitoneum | Normal |
| **UPS15** | Retroperitoneum | Gain |
| **UPS6** | Lower extremity | Normal |
| **UPS14** | Trunk | NA |
| **LMS6** | Retroperitoneum | Gain |
| **LMS16** | Retroperitoneum | Gain |
| **LMS22** | Retroperitoneum | Gain |
| **LMS15** | Lower extremity | Normal |
| **LMS20** | Lower extremity | Normal |
| **LMS5** | Trunk | Normal |
